# Novel Compound Heterozygous Variants in the *TCTN2* Gene Causing Meckel–Gruber Syndrome 8 in a Non‐Consanguineous Chinese Family

**DOI:** 10.1002/mgg3.70160

**Published:** 2025-11-29

**Authors:** Qi Yang, Wei He, Qiang Zhang, Sheng Yi, Xunzhao Zhou, Shujie Zhang, Shang Yi, Qinle Zhang, Jingsi Luo

**Affiliations:** ^1^ Guangxi Key Laboratory of Birth Defects Research and Prevention, Guangxi Key Laboratory of Reproductive Health and Birth Defects Prevention Maternal and Child Health Hospital of Guangxi Zhuang Autonomous Region Nanning China; ^2^ Department of Genetic and Metabolic Central Laboratory Maternal and Child Health Hospital of Guangxi Zhuang Autonomous Region Nanning China; ^3^ Guangxi Clinical Research Center for Birth Defects Maternal and Child Health Hospital of Guangxi Zhuang Autonomous Region Nanning China; ^4^ Prenatal Diagnosis Center Maternal and Child Health Hospital of Guangxi Zhuang Autonomous Region Nanning China; ^5^ Guangxi Clinical Research Center for Pediatric Diseases Maternal and Child Health Hospital of Guangxi Zhuang Autonomous Region Nanning China

**Keywords:** MKS8, novel variant, prenatal ultrasound, *TCTN2*, whole‐exome sequencing

## Abstract

**Introduction:**

Meckel‐Gruber syndrome (MKS, OMIM 24,900), also known as Meckel syndrome, is a rare and severe autosomal recessive disorder. The syndrome is typically characterized by a triad of occipital encephalocele, bilateral renal cystic dysplasia, and postaxial polydactyly. MKS shows significant clinical heterogeneity, which poses challenges for accurate prenatal diagnosis. Prenatal ultrasound is an important tool for detecting potential cases, but the complexity of MKS often requires additional advanced techniques such as prenatal whole‐exome sequencing (WES) to provide more accurate molecular genetic evidence.

**Methods:**

In this study, we used whole‐exome sequencing (WES) to analyze the genetic causes of suspected MKS in a Chinese fetus. Sanger sequencing was used to confirm the origin of the variants. The classification of variants was carried out in accordance with the guidelines of the American College of Medical Genetics and Genomics/Association for Molecular Pathology (ACMG/AMP).

**Results:**

A 26‐year‐old pregnant woman was referred to our antenatal centre for genetic diagnosis at 13 + 5 weeks of gestation due to fetal occipital encephalocele and renal cysts detected by ultrasound. Two novel heterozygous variants, c.1047delA (p.Val351fs*1) and c.1336C>T (p.Arg446*), were identified in *TCTN2*. Sanger sequencing revealed that the c.1047delA (p.Val351fs*1) variant was inherited from the mother and the c.1336C>T (p.Arg446*) variant was inherited from the father. According to the ACMG/AMP guidelines, these two variants were evaluated as pathogenic.

**Conclusions:**

This study further expands the genetic mutation spectrum of *TCTN2* and is conducive to further clarifying the relationship between the genotype and phenotype of MKS8. Severe variants in the *TCTN2* gene appear to be more likely to lead to MKS8. Clinically, the triad is an important basis for the diagnosis of MKS8, while other variable phenotypes of MKS8 can provide additional information for prenatal diagnosis. The combination of prenatal ultrasound and WES can provide a more comprehensive and accurate diagnosis of MKS8, which will greatly aid support for early intervention and treatment.

## Graphical abstract

Genetic analysis of the fetus with Meckel–Gruber syndrome 8 and the spectrum of *TCTN2* variants.

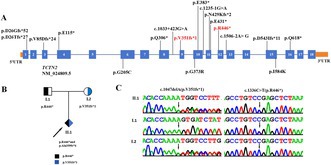

## 1 Introduction

The ciliopathies are a group of highly heterogeneous inherited disorders and are caused by defects in cilia development and function. As the most serious ciliopathies, Meckel‐Gruber syndrome (MKS, OMIM 24,900) has been classified into at least 14 types (MKS1~14), caused by variants in genes such as *MKS1*, *TMEM216*, *TMEM67*, *CEP290*, *RPGRIP1L*, *CC2D2A*, *NPHP3*, *TCTN2*, *B9D1*, *B9D2*, *TMEM231*, *Kif14*, *TMEM107*, *TXNDC15* (Hartill et al. 2017; Parisi 2019). Meckel‐Gruber syndrome 8 is a rare fatal autosomal recessive disorder characterized by occipital encephalocele, polycystic kidneys, and polydactyly. It is caused by homozygous or compound heterozygous variants in the tectonic family member 2 (*TCTN2*, NM_024809.5, MIM 613846) (Shaheen et al. 2011). Mutations in the *TCTN2* gene are responsible for roughly 1% of all instances of Meckel‐Gruber syndrome (Litz Philipsborn et al. 2021). The ciliary transition zone (TZ) serves as a critical gatekeeper for the cilium, regulating the entry of proteins, lipids, and other signaling components into this organelle (Reiter et al. 2012). This regulatory function is essential for the proper growth and extension of the cilium, ensuring the integrity of the signaling microenvironment within (Chih et al. 2011; Kanamaru et al. 2022; Schou et al. 2017; Li et al. 2016). The TZ prevents the entry of extraneous or potentially deleterious molecules that could disrupt the signaling cascade, thereby maintaining the fidelity of intraciliary signaling. For example, in the context of the Hedgehog (Hh) signaling pathway, the TZ's gating role is particularly crucial. It ensures that the Smoothened (SMO) protein enters the cilium at the appropriate time and in the correct conformation to activate downstream signaling (Li et al. 2016; Schembs et al. 2022; Tang and Cheng 2014). This precise control is vital for cell fate determination and other physiological processes. Specifically, loss‐of‐function mutations in *TCTN2*, a component of the ciliary transition zone, result in reduced ciliogenesis and impaired Hh signaling (Abrams and Reiter 2021; Wang et al. 2017). Furthermore, TCTN2 inhibits neuronal apoptosis by promoting autophagy (Ren et al. 2019). Consequently, loss‐of‐function mutations in the *TCTN2* gene result in defects in ciliary biogenesis and function, leading to a wide range of developmental defects.

**FIGURE 1:**
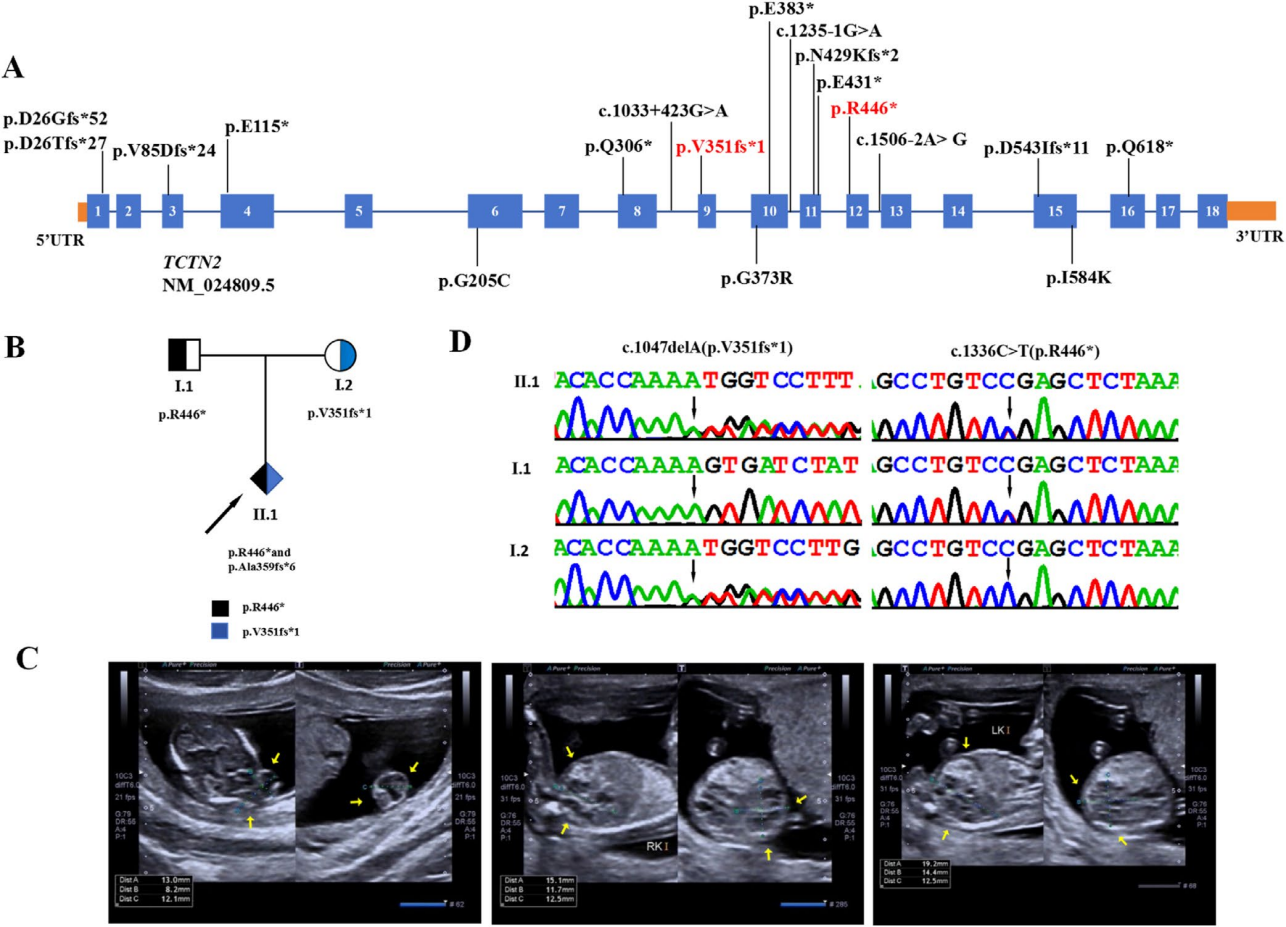
Clinical and genetic features. (A) The spectrum of pathogenic variants in *TCTN2*. The red variants represent the novel variants identified in this study. (B) Pedigree chart of the family of the fetus with Meckel–Gruber syndrome 8. The proband is indicated by a black arrow. (C) The patient's prenatal ultrasound at 13 weeks and 5 days' gestation showed occipital brain enlargement and markedly enlarged echogenic kidneys with polycystic changes. (D) Sanger sequencing DNA chromatograms of *TCTN2* indicating the frameshift c.1047delA (p.Val351fs*1) variant inherited from the mother and the nonsense variant c.1336C>T (p.Arg446*) was transmitted by the father.

To date, only seven variants in the *TCTN2* gene have been reported in patients with Meckel‐Gruber syndrome 8 (Shaheen et al. 2011, 2016; Litz Philipsborn et al. 2021; Zhang et al. 2020; Al‐Hamed et al. 2016; Sang et al. 2011). The seven variants identified include one founder splicing variant, three nonsense variants and three frameshift variants (Table 1 and Figure 1A). There is currently no complete understanding of how different variants in the same *TCTN2* gene cause distinct clinical phenotypes. Additional reports on *TCTN2* variants and their respective phenotypes will facilitate a better understanding of this disease and an exploration of the relationship between genotype and phenotype. Here, we described a fetus from a non‐consanguineous Chinese family presenting with meningoencephalocele and enlarged kidneys with multiple cystic changes. By whole‐exome sequencing (WES), we identified c.1047delA (p.Val351fs*1) and c.1336C>T (p.Arg446*) compound heterozygous variants in the *TCTN2* gene. In addition, through a review of the literature, we have summarized the genotypic, phenotypic and clinical features of Meckel‐Gruber syndrome 8.

**TABLE 1:** Summary of reported information on clinical phenotypes in association with *TCTN2* variants in patients with MKS8.

Patients	Our patient	Zhang et al.	Philipsborn et al.	Shaheen et al.				
Clinical data	Proband	1 Patient	4 Patients	One family (4 patients)	2 Patients	8 Patients	1 Patient	3 Patients
TCTN2 (NM_024809.5) variants	c.1047delA (p.V351fs*1) and c.1336C>T (p.R446*)	c.343G>T (p.E115*) and c.1540C>T (p.Q514*)	c.1506‐2A>G	c.1506‐2A>G	c.1286dupA (p.N29Kfs*2)	c.1506‐2A>G	c.254_255delTG (p.V85Dfs*24)	c.1852C>T (p.Q618*)
Renal cystic disease	+	+	4/4	4/4	1/1	8/8	+	3/3
Occipital encephalocele	+	+	4/4	4/4	2/2	8/8		
Polydactyly	−	+	1/4	4/4	2/2	7/7		
Cardiovascular	−	+		2/4				
Other structural brain abnormalities	−		4/4					
Hydramnios/oligohydramnios	−	−	2/4			4/4		
Increased nuchal translucency	−		2/4					
Intrauterine growth retardation			1/4					
Potterlike facies						2/2		
Microcephaly	−	−			1/1	1/1		
Cleft lip/palate	−	−	1/4	1/4		2/2		
Prominent forehead		−						
Low‐set/malformed ears		+	1/4		1/1	4/4		
Short neck		−			1/1	3/3		
Micrognathia		+	1/4					1/1
Hypoplastic nose			1/4			3/3		
Ophthalmic dysplasia			1/4			2/2		
Sloping forehead		−				2/2		
Short bowed long bones	−	−	2/4			1/1		
Narrow chest	−	−			1/1	1/1		
Clubfeet	−	−	2/4	1/4		3/3		
Pulmonary hypoplasia			1/4			1/1		
Abnormality of the bladder			2/4					
Ambiguous genitalia			1/4					

## 2 Material and Methods

### 2.1 Subjects and Ethics Approval

The study was approved by the Institutional Review Board and Ethics Committee of Guangxi Maternal and Child Health Hospital (GXMC20240123), and detailed informed consent was obtained from the patient's parents for the publication of the patient's relevant clinical data in this paper.

### 2.2 Prenatal Ultrasonography

Patients underwent ultrasound screening utilizing a 3–6 MHz transabdominal probe on a Toshiba Aplio500 color Doppler ultrasound diagnostic machine (Toshiba, Tokyo, Japan). The ultrasound settings for pregnancy screening were precisely adjusted in accordance with the manufacturer's recommended parameters. A highly experienced ultrasound physician with over five years of expertise in fetal ultrasound assessment carried out the ultrasound screening. Furthermore, two consultant ultrasound physicians performed the measurements, and each measurement was meticulously checked three times by one examiner for accuracy. The final data were recorded as the average values of these measurements.

### 2.3 Genetic Analysis

The QIAamp DNA Mini Kit Tissue kit (Qiagen, Germany) used to obtain Fetal DNA was extracted from chorionic villus sample. Genomic DNA was isolated from peripheral blood lymphocytes collected from all subjects using the La‐Aid DNA kit (Zeesan Biotech Co. Ltd., Xiamen, China). For WES, genomic DNA was used to generate exomic libraries using the Agilent SureSelect Human All Exon V6 Kit (Agilent Technologies, Santa Clara, CA), following the manufacturer's sample preparation protocol. After sequencing on the Illumina HiSeq2500 platform (Illumina, San Diego, CA), the reads generated by the BWA package (v. 0.7.15) were aligned to the human reference genome (hg19/RCh37). Subsequently, the TGex software (LifeMap Sciences Inc. v5.7) and the Genome Analysis Toolkit (GATK) were used for variant calling and variant annotation. Mutation Taster (http://www.mutationtaster.org), CADD (https://cadd.gs.washington.edu/snv), SIFT (http://sift.jcvi.org/), PROVEAN (http://www.provean.jcvi.org/) and PolyPhen 2.0 (http://genetics.bwh.harvard.edu/pph2/) were used to predict the pathogenicity of candidate variants and for prioritization of variants. Sanger sequencing was used to validate the variant in proband and unaffected family members. The following primers designed by primer3 were used: 5′‐TACTTTGAGTAAGTGTGATCATGGTA‐3′, and 5′‐GGACAGAACGAGGAACAAATG‐3′ for c.1047delA (p.Val351fs*1); and5′‐CCGAAGTTGCCATCTTTCTC‐3′, and 5′‐ATTTGCCAAAGGGCTAATCA‐3′ for c.1336C>T (p.Arg446*). The classification and interpretation of the identified variants followed the guidelines of the American College of Medical Genetics and Genomics (ACMG) (Richards et al. 2015; Miller et al. 2023).

## 3 Results

### 3.1 Clinical Phenotype

A 26‐year‐old healthy woman (gravida 1 para 0) was referred to the Guangxi Zhuang Autonomous Region Maternal and Child Healthcare Hospital (Nanning, China) at 13 + 5 weeks' gestation for ultrasound findings of fetal brain malformation (Figure 1B). The couple was non‐consanguineous and had no family history of neurological or renal disorders. The pregnancy was under medical supervision. No medication was taken during the pregnancy. Prenatal ultrasound showed an occipital brain bulge and grossly enlarged echogenic kidneys; however, the amniotic fluid was in a normal state and no skeletal abnormalities were observed (Figure 1C). NT performed at 12 weeks of gestation was normal (2 mm).

### 3.2 Genetic Analysis

Whole‐exome sequencing (WES) was used to identify potential gene mutations in the fetal. WES sequencing generated 8.46 Gb of data, with an adequate coverage rate of 99.3% in the target regions and a 20‐fold coverage depth for 99.2% of the target regions. A total of 118,656 single nucleotide variants (SNVs) or insertion–deletion variants (indels) were detected in the coding regions and splice sites (splice junctions of 10 bp in length). Data filtering excluded variants with a minor allele frequency (MAF) greater than 1% in gnomAD, dbSNP132, ESP, 1000G and our in‐house database, yielding 819 unique single nucleotide polymorphisms (SNPs). After further exclusion of possible benign variants (including synonymous variants and missense variants predicted to be harmless by in silico prediction tools), 561 variants remained. After data analysis using TGex analysis software (https://tgex.genecards.cn/), we extracted eight variants from seven genes (*PKD1*, *TCTN2*, *FRAS1*, *GUCY2D*, *GLI3*, *CHRM3* and *FRMPD4*) that matched the known phenotypes. The variants in the *FRAS1* and *CHRM3* genes were heterozygous. As the diseases caused by mutations in these genes follow an autosomal recessive inheritance pattern, these genes were excluded. For *PKD1*, *GUCY2D*, *GLI3* and *FRMPD4* genes, their mutations were inherited from unaffected parents, further confirming that they were not the pathogenic factors for the observed phenotypes. Then, we identified two heterozygous *TCTN2* variants, c.1047delA (p.Val351fs*1) and c.1336C>T (p.Arg446*), that were identified in the proband (Figure 1B). Further analysis through Sanger sequencing showed that the father had the heterozygous c.1047delA (p.Val351fs*1) variant while the mother had the heterozygous c.1336C>T (p.Arg446*) variant (see Figure 1D).

## 4 Discussion

Meckel‐Gruber syndrome 8 (MKS8) is a rare, lethal autosomal‐recessive genetic disease. In 2011, Shaheen first described this disease in a consanguineous family of Arab origin. Patients with MKS8 in this family all exhibited polycystic kidneys, occipital encephalocele and postaxial polydactyly, and died during the perinatal period (Shaheen et al. 2011). It has been identified that a splicing variant in the *TCTN2* gene is the cause of these symptoms in these patients. Subsequently, MKS8 cases with pathogenic mutations in this gene were identified in six families from around the world by whole‐exome sequencing (WES) (Shaheen et al. 2011; Litz Philipsborn et al. 2021; Schou et al. 2017; Li et al. 2016; Schembs et al. 2022; Tang and Cheng 2014). In this study, we identified compound heterozygous variants (c.1047delA (p.Val351fs*1) and c.1336C>T (p.Arg446*)) in the *TCTN2* gene (NM_024809.5) in a fetus through whole‐exome sequencing. The c.1047delA (p.Val351fs*1) variant was not found in the Human Gene Mutation Database (http://www.hgmd.cf.ac.uk/ac/), HPSD (http://liweilab.genetics.ac.cn/HPSD/), dbSNP (http://www.ncbi.nlm.nih.gov/SNP/), ExAC, and gnomAD (https://gnomad.broad institute.org/). It is located in the ninth exon of the *TCTN* gene and causes a premature termination codon, resulting in loss of function. MutationTaster predicted that c.1047delA (p.Val351fs*1) is disease‐causing. The other *TCTN2* variant, c.1336C>T (p.Arg446*), is present in the Genome Aggregation Database (gnomAD v.2.1.1) with a minor allele frequency of 0.0000014. This variant is located in the twelfth exon of the *TCTN2* gene and also causes a premature termination codon, leading to loss of function. The variant of c.1336C>T (p.Arg446*) was also predicted to be disease‐causing by MutationTaster. According to the criteria and guidelines of the ACMG/AMP for the interpretation of sequence variants, c.1047delA (p.Val351fs*1) was assessed to be pathogenic (PVS1, PM2, PP4), and c.1336C>T (p.Arg446*) was assessed to be pathogenic (PVS1, PM2, PM3, PP4).

The clinical features and molecular information of patients with Meckel‐Gruber syndrome 8, including the present study, are summarized in Table 1. The most striking feature of the disease is the ‘triad’ of renal cysts (23/23), occipital craniosynostosis (20/20) and polydactyly (15/18) in almost all patients. Notably, polydactyly was not found in this case at 13 weeks' gestation. Dysmorphic facial features were observed in eight patients. These facial deformities included low‐set/malformed ears, micrognathia, nasal hypoplasia, ocular hypoplasia and a sloping forehead. Other variable phenotypes included heart defects observed in 3 patients, pulmonary dysplasia in 2 patients, increased nuchal translucency in 2 patients, bladder abnormalities in 2 patients, intrauterine growth retardation in 1 patient, ambiguous genitalia in 1 patient, other structural brain abnormalities in 4 patients, and other skeletal malformations in 8 patients (including chest constriction, short and long bone disorders and talipes equinovarus). These variable malformations may become more pronounced and complex as the weeks of pregnancy increase. Although these malformations are not detected by ultrasound at 13 weeks of gestation, the fetus in our study may develop various other malformations as the gestational weeks progress. However, the pregnant woman opted to terminate the pregnancy at 15 weeks, and no such abnormalities were detected in the post‐procedure examinations. Furthermore, these variable dysmorphic features not only provide additional information for early diagnosis by ultrasound but also pose challenges.

We also summarized the *TCTN2* mutations reported so far in patients with Meckel–Gruber syndrome (MKS) and Joubert syndrome (JS) (Table 2, Figure 1A). All of the variants are located in the extracellular domain. Among the 19 pathogenic variants, 11 are associated with JS patients, while 9 are related to MKS patients (Shaheen et al. 2011, 2016; Litz Philipsborn et al. 2021; Schou et al. 2017; Li et al. 2016; Schembs et al. 2022; Tang and Cheng 2014; Wang et al. 2017; Ren et al. 2019; Zhang et al. 2020; Al‐Hamed et al. 2016; Sang et al. 2011). There does not seem to be an obvious correlation between the genotype and the phenotype in *TCTN2*. Nonsense variants or frameshift variants in either homozygous or compound heterozygous states have been detected in both Joubert syndrome type 24 patients and MKS8 patients. However, all variants found in MKS8 patients were severe forms of mutations (nonsense mutations, frameshift mutations and splice site mutations) and it appears that these null mutations are more likely to lead to more severe manifestations.

**TABLE 2:** *TCTN2* gene variants associated with Meckel‐Gruber syndrome 8 or Joubert syndrome type 24.

CDS position^a^	Protein change	Affected exon/intron	Clinical significance	Associated diseases	References
c.1047delA	p.V351fs*1	Exon 9	Pathogenic	Meckel‐Gruber syndrome 8	This study
c.1336C>T	p.R446*	Exon 12	Pathogenic	Meckel‐Gruber syndrome 8	This study Sang et al. (2011)
c.343G>T	p.E115*	Exon 4	Pathogenic	Meckel‐Gruber syndrome 8	Zhang et al. (2020)
c.1540C>T	p.Q514*	Exon 14	Pathogenic	Meckel‐Gruber syndrome 8	Zhang et al. (2020)
c.1506‐2A>G		Intron 13	Pathogenic	Meckel‐Gruber syndrome 8	Litz Philipsborn et al. (2021), Shaheen et al. (2011), Shaheen et al. (2016)
c.1286dupA	p.N429Kfs*2	Exon11	Pathogenic	Meckel‐Gruber syndrome 8	Shaheen et al. (2016)
c.254_255delTG	p.V85Dfs*24	Exon 3	Pathogenic	Meckel‐Gruber syndrome 8	Al‐Hamed et al. (2016)
c.1852C>T	p.Q618*	Exon 16	Pathogenic	Meckel‐Gruber syndrome 8	Al‐Hamed et al. (2016)
c.76dupG	p.D26Gfs*52	Exon 1	Pathogenic	Meckel‐Gruber syndrome 8 or Joubert syndrome type 24	Sang et al. (2011), Bachmann‐Gagescu et al. (2015)
c.1117G>A	p.G373R	Exon 10	Likely pathogenic	Joubert syndrome type 24	Sang et al. (2011), Juric‐Sekhar et al. (2012)
c.1626delT	p.D543Ifs*11	Exon 15	Pathogenic	Joubert syndrome type 24	Bachmann‐Gagescu et al. (2015)
c.613G>T	p.G205C	Exon 6	Likely pathogenic	Joubert syndrome type 24	Bachmann‐Gagescu et al. (2015)
c.1751T>A	p.I584K	Exon 15	Likely pathogenic	Joubert syndrome type 24	Bachmann‐Gagescu et al. (2015)
c.1291G>T	p.E431*	Exon 11	Pathogenic	Joubert syndrome type 24	Bachmann‐Gagescu et al. (2015)
c.916 C>T	p.Q306*	Exon 8	Pathogenic	Joubert syndrome type 24	Fang et al. (2023), Hiraide et al. (2023)
c.1147G>T	p.E383*	Exon 10	Pathogenic	Joubert syndrome type 24	Fang et al. (2023)
c.1235‐1G>A		Intron 10	Pathogenic	Joubert syndrome type 24	Huppke et al. (2015)
c.76delG	p.D26Tfs*26	Exon 1	Pathogenic	Joubert syndrome type 24	Juric‐Sekhar et al. (2012), Bachmann‐Gagescu et al. (2015)
c.1033+423G>A		Intron 8	Likely pathogenic	Joubert syndrome type 24	Hiraide et al. (2023)

^a^ All cDNA and protein positions refer to the NM_024809.5 *TCTN2* transcript.

The mechanism by which loss of function of the *TCTN2* gene leads to Meckel‐Gruber syndrome type 8 remains to be further investigated. The wide expression pattern of TCTN2 in human tissues suggests that mutations in the *TCTN2* gene are likely to be the cause of the extensive and heterogeneous phenotypes of Meckel syndrome type 8. TCTN2 has been shown to form the MKS complex with multiple ciliopathic proteins that are associated with Meckel and Joubert syndromes, including MKS1, TMEM216, TMEM67, CEP290, CC2D2A, B9D1, TCTN1, and TCTN3 (Garcia‐Gonzalo et al. 2011). This complex has been identified as playing a crucial role in maintaining the structural integrity and ensuring the proper functionality of the ciliary transition zone. Studies have revealed that the loss of any single component, including TCTN2, causes tissue‐specific defects in ciliogenesis and ciliary membrane composition (Reiter et al. 2012; Garcia‐Gonzalo et al. 2011; Sang et al. 2011; Dowdle et al. 2011; Yee et al. 2015). Furthermore, since primary cilia are found on almost every mammalian cell, the disruption of TCTN2—which acts as a gatekeeper regulating the entry and exit of proteins from cilia—can lead to disordered protein trafficking in and out of cilia, consequently resulting in abnormalities in multiple organ systems such as the brain, kidney, bone, liver, heart, digestive system, and reproductive system (Weng et al. 2018; Tang and Cheng 2014; Bachmann‐Gagescu et al. 2015; Fang et al. 2023; Huppke et al. 2015). Moreover, TCTN2 exerts a pivotal regulatory function within the Shh and Hh signalling pathways, predominantly through its action within the ciliary transition zone (TZ) (Weng et al. 2018; Abrams and Reiter 2021; Wang et al. 2017; Safavian et al. 2023). Primary cilia function as cellular antennae that detect and transduce extracellular signals, including those of the Hh pathway, by concentrating and organising signal transduction components (Jing et al. 2023). Specifically, TCTN2 interacts with other TZ proteins to form a complex that regulates ciliary membrane composition and Smoothened (SMO) transport (Chandra et al. 2022). When the Shh ligand binds to the Patched (PTCH) receptor, SMO inhibition is released, allowing SMO to enter the cilium (Bangs and Anderson 2017). The process is facilitated by the internal ciliary transport (IFT) proteins, which are indispensable for the transportation of signaling molecules along the cilium (Gigante and Caspary 2020). TCTN2 activates the downstream signalling cascade response by ensuring the correct positioning of SMO within the cilium, which in turn activates the Gli transcription factor (Wang et al. 2017). When the transmembrane domain of TCTN2 is deleted or its key sites are altered, this can disrupt the protein network to varying degrees and then induce the occurrence of Meckel syndrome (MKS) or Joubert syndrome (JS) type 24 depending on the responses of different downstream elements. Further functional studies of these variants will help us gain a more comprehensive understanding of Joubert syndrome type 24 and MKS8 and to explore their underlying mechanisms in depth.

## 5 Conclusion

In conclusion, we identified a novel compound heterozygous variant of the TCTN2 gene in a fetus diagnosed with MSK8. This is the first report of the c.1047delA (p.Val351fs*1) and c.1336C>T (p.Arg446*) variants in the *TCTN2* gene, thereby expanding the variant spectrum of TCTN2‐associated MKS8. Additionally, the diverse phenotypes of the patient described in detail in this study also contribute to the understanding of the phenotypic spectrum of MKS8. The triad is an important basis for the diagnosis of MKS8, and the clinical heterogeneity of MKS8 provides additional information for prenatal diagnosis. The combination of prenatal ultrasound and whole‐exome sequencing (WES) provides a more comprehensive and accurate diagnosis of MKS8, contributing to personalized medicine and targeted treatment strategies. Future research efforts should focus on further elucidating the underlying molecular mechanisms and developing more effective diagnostic and therapeutic interventions for this debilitating syndrome.

## Author Contributions

Qi Yang and Jingsi Luo designed and drafted the manuscript. Wei He, Qiang Zhang, Xunzhao Zhou, Sheng Yi, Shujie Zhang, Shang Yi and Qinle Zhang collected the patients' clinical information and analyzed the WES data. Qi Yang and Wei He revised the manuscript. All authors contributed to the coordination of the study and revised the manuscript. All authors read and approved the final version of the manuscript.

## Funding

This research was supported by the Health Department of Guangxi Province (Grant No. Z‐A20220256).

## Ethics Statement

This study was approved by the Institutional Review Board and Ethics Committee of Guangxi Maternal and Child Health Hospital, and adhered to the principles delineated in the Declaration of Helsinki. All of the methodologies have been rigorously implemented in accordance with the applicable guidelines and regulations. Written informed consent was obtained from the patients or their next of kin/legal guardian for the publication of any potentially identifiable images or data included in this article.

## Conflicts of Interest

The authors declare no conflicts of interest.

## Data Availability

All data that support the findings of the current study are available from the corresponding author upon reasonable request.
